# Draft Genome Sequence of Clostridium estertheticum-Like Strain FP3, Isolated from Spoiled Uncooked Lamb

**DOI:** 10.1128/MRA.00434-20

**Published:** 2020-05-14

**Authors:** Nikola Palevich, Faith P. Palevich, Paul H. Maclean, Ruy Jauregui, Eric Altermann, John Mills, Gale Brightwell

**Affiliations:** a AgResearch Limited, Grasslands Research Centre, Palmerston North, New Zealand; b Riddet Institute, Massey University, Palmerston North, New Zealand; University of Rochester School of Medicine and Dentistry

## Abstract

Clostridium estertheticum-like strain FP3 was isolated from vacuum-packaged refrigerated spoiled lamb. This bacterium is psychrotrophic, Gram positive, spore-forming, and a strict anaerobe. Here, we report the generation and annotation of the 5.6-Mb draft genome sequence of *C. estertheticum*-like strain FP3.

## ANNOUNCEMENT

Several bacterial species of the genus *Clostridium* have been recognized in occurrences of blown-pack spoilage (BPS), predominantly *C. estertheticum* (1). The potential of a BPS episode is one of the primary reasons that abattoirs cannot adopt more cost-effective techniques that would provide substantial improvements in quality meat production. Clostridium estertheticum-like strain FP3 is a Gram-positive, spore-forming, and slow-growing psychrotrophic anaerobe, originally isolated from vacuum-packaged refrigerated spoiled lamb at AgResearch Ltd., Palmerston North, New Zealand. Existing molecular-based detection strategies grouped FP3 into the nontoxigenic *C. estertheticum*-like cluster with >89% similarity based on recent amplified rDNA (ribosomal DNA) restriction analysis (ARDRA) of psychrotolerant *Clostridium* isolates (2), in addition to a positive test with the real-time *C. estertheticum*-like-specific PCR assay (3).

Strain FP3 was isolated in 2017 from the meat drip of a fully distended pack of vacuum-packaged lamb in which the meat was discolored (gray-brown). FP3 was cultured anaerobically from the meat drip at 10°C in 10-fold suspensions of prereduced peptone-yeast extract-glucose-starch (PYGS) broth (3). Genomic DNA was extracted using a modified phenol-chloroform procedure as previously described (4–6). Genomic DNA was prepared for whole-genome sequencing using an Illumina TruSeq Nano library preparation kit (Illumina, Inc., San Diego, CA) according to the manufacturer’s instructions, and sequencing was performed using a MiSeq instrument (Illumina, Inc.) with 250-bp paired-end sequencing. In total, 3,189,796 raw reads were generated. Trimming and *de novo* assembly were performed using the A5-miseq pipeline v20169825 (7), with QUAST v5.0.0 (8) used to assess the assembly scaffold quality, such as the number of contigs, G+C content, *N*_50_ value, and total size. The result was a 5,555,543-bp draft genome assembly with 85 contigs, an average coverage of 139×, an *N*_50_ value of 245,874 bp, with the largest scaffold being 919,536 bp, and a G+C content of 31.5%.

The FP3 draft genome was initially annotated using the GAMOLA2 (9) and OmicsBox v1.1.164 (10) software packages, with the NCBI Prokaryotic Genome Annotation Pipeline (PGAP) software package (11) used to generate the final annotation. Default parameters were used for all software unless otherwise specified. Annotated features include 5,023 putative protein-coding genes (PCGs) with 101 tRNAs, 60 rRNAs, and 150 noncoding RNA (ncRNA) elements. Functional annotation of the carbohydrate-active enzymes (12) implies that different members of the Clostridium estertheticum species vary in their ability to utilize simple carbohydrates for growth, especially for complex polysaccharides. dbCAN2 (13) predicted 48 glycoside hydrolases (GHs), 30 glycosyl transferases (GTs), 5 carbohydrate esterases (CEs), and 9 carbohydrate-binding protein module (CBM) families but no polysaccharide lyases (PLs). Interestingly, FP3 encodes half as many CBMs and approximately 15 fewer GHs than the closely related *C. estertheticum* type strains (5, 14). In conclusion, this draft genome sequence of *C. estertheticum*-like strain FP3 will facilitate future functional genomic studies investigating the bacterial genetic mechanisms associated with BPS.

### Data availability.

The project data for Clostridium estertheticum-like strain FP3 have been submitted under GenBank accession number JAAMNH000000000 and BioProject accession number PRJNA574489 and the raw sequences under Sequence Read Archive (SRA) accession number SRR11113221.

## References

[1] Mills J, Donnison A, Brightwell G 2014 Factors affecting microbial spoilage and shelf-life of chilled vacuum-packed lamb transported to distant markets: a review. Meat Sci 98:71–80. doi:10.1016/j.meatsci.2014.05.002. 24875594

[2] Brightwell G, Horváth KM 2018 Molecular discrimination of New Zealand sourced meat spoilage associated psychrotolerant Clostridium species by ARDRA and its comparison with 16s RNA gene sequencing. Meat Sci 138:23–27. doi:10.1016/j.meatsci.2017.12.007. 29289715

[3] Brightwell G, Clemens R 2012 Development and validation of a real-time PCR assay specific for *Clostridium estertheticum* and *C. estertheticum*-like psychrotolerant bacteria. Meat Sci 92:697–703. doi:10.1016/j.meatsci.2012.06.025. 22782010

[4] Bouillaut L, McBride SM, Sorg JA 2011 Genetic manipulation of *Clostridium difficile*. Curr Protoc Microbiol Chapter 9:Unit 9A.2. 10.1002/9780471729259.mc09a02s20 PMC3615975 21400677

[5] Palevich N, Palevich FP, Maclean PH, Jauregui R, Altermann E, Mills J, Brightwell G 2019 Draft genome sequence of *Clostridium estertheticum* subsp. *laramiense* DSM 14864^T^, isolated from spoiled uncooked beef. Microbiol Resour Announc 8:e01275-19. doi:10.1128/MRA.01275-19. 31753947 PMC6872889

[6] Palevich N, Palevich FP, Maclean PH, Jauregui R, Altermann E, Mills J, Brightwell G 2020 Draft genome sequence of psychrotolerant *Clostridium* sp. strain M14, isolated from spoiled uncooked venison. Microbiol Resour Announc 9:e00314-20. doi:10.1128/MRA.00314-20. 32299886 PMC7163024

[7] Coil D, Jospin G, Darling AE 2015 A5-miseq: an updated pipeline to assemble microbial genomes from Illumina MiSeq data. Bioinformatics 31:587–589. doi:10.1093/bioinformatics/btu661. 25338718

[8] Gurevich A, Saveliev V, Vyahhi N, Tesler G 2013 QUAST: quality assessment tool for genome assemblies. Bioinformatics 29:1072–1075. doi:10.1093/bioinformatics/btt086. 23422339 PMC3624806

[9] Altermann E, Lu J, McCulloch A 2017 GAMOLA2, a comprehensive software package for the annotation and curation of draft and complete microbial genomes. Front Microbiol 8:346. doi:10.3389/fmicb.2017.00346. 28386247 PMC5362640

[10] Conesa A, Götz S, García-Gómez JM, Terol J, Talón M, Robles M 2005 Blast2GO: a universal tool for annotation, visualization and analysis in functional genomics research. Bioinformatics 21:3674–3676. doi:10.1093/bioinformatics/bti610. 16081474

[11] Tatusova T, DiCuccio M, Badretdin A, Chetvernin V, Nawrocki EP, Zaslavsky L, Lomsadze A, Pruitt KD, Borodovsky M, Ostell J 2016 NCBI Prokaryotic Genome Annotation Pipeline. Nucleic Acids Res 44:6614–6624. doi:10.1093/nar/gkw569. 27342282 PMC5001611

[12] Cantarel BL, Coutinho PM, Rancurel C, Bernard T, Lombard V, Henrissat B 2009 The Carbohydrate-Active EnZymes database (CAZy): an expert resource for glycogenomics. Nucleic Acids Res 37:D233–D238. doi:10.1093/nar/gkn663. 18838391 PMC2686590

[13] Zhang H, Yohe T, Huang L, Entwistle S, Wu P, Yang Z, Busk PK, Xu Y, Yin Y 2018 dbCAN2: a meta server for automated carbohydrate-active enzyme annotation. Nucleic Acids Res 46:W95–W101. doi:10.1093/nar/gky418. 29771380 PMC6031026

[14] Yu Z, Gunn L, Brennan E, Reid R, Wall PG, Gaora PÓ, Hurley D, Bolton D, Fanning S 2016 Complete genome sequence of *Clostridium estertheticum* DSM 8809, a microbe identified in spoiled vacuum packed beef. Front Microbiol 7:1764. doi:10.3389/fmicb.2016.01764. 27891116 PMC5104964

